# Association between seasons with substantial atmospheric pressure change and migraine occurrence: a retrospective cohort study using Japanese claims data and meteorological data

**DOI:** 10.3389/fneur.2025.1600822

**Published:** 2025-09-10

**Authors:** Muneto Tatsumoto, Koichi Hirata, Takeo Nakayama, Kentaro Yamato, Norihiro Nakamichi, Lyo Inuyama

**Affiliations:** ^1^Canon Marketing Japan Inc., Tokyo, Japan; ^2^Department of Neurology, Dokkyo Medical University, Mibu, Japan; ^3^Department of Health Informatics, School of Public Health, Kyoto University, Kyoto, Japan; ^4^Department of Public Health, Graduate School of Medicine, Juntendo University, Tokyo, Japan; ^5^Department of Medical Affairs, Otsuka Pharmaceutical Co., Ltd., Tokyo, Japan

**Keywords:** migraine, atmospheric pressure change, season, weather, claims database

## Abstract

**Background:**

This study investigated the impact of seasons with substantial atmospheric pressure change on the occurrence of migraine using large-scale data from a Japanese health insurance claims database matched with meteorological data.

**Methods:**

This retrospective cohort study used JMDC Claims Data and national meteorological data from Japan. Patients with a diagnostic record of migraine were included and categorized into eight regional subgroups based on the location of the healthcare facility at which they were initially diagnosed with migraine. The time to migraine occurrence, defined as the duration from the first day of each season to the prescription of triptans, was compared between the seasons with the highest and lowest atmospheric pressure change.

**Results:**

A total of 26,777 individuals were included in this study. Summer showed the lowest atmospheric pressure change across all eight regions. Conversely, winter showed the highest atmospheric pressure change in seven regions, while autumn showed the highest change in one region. No differences were observed in survival curves between the seasons with the highest and lowest atmospheric pressure change in any region. In Cox regression analysis, hazard ratio for the season with the highest atmospheric pressure change was 0.970 (95% CI: 0.951–0.989) in the minimally adjusted model, which included sex and age. Conversely, the fully adjusted model, which incorporated eight covariates, indicated a hazard ratio of 1.294 (95% CI: 1.007–1.663) for the season with the highest atmospheric pressure change.

**Conclusion:**

This study, using a large Japanese administrative claims database, did not identify a significant association between seasons with substantial atmospheric pressure change and migraine occurrence. Future research should consider examining more granular residential data, beyond the prefecture level, for a more detailed analysis.

## Introduction

Migraine is a common condition in the general population, and leads to a significant burden and reduction in quality of life. The prevalence of migraine in Japan has been reported to range from 3.2 to 8.4% (1–3). Migraine is more prevalent in women (1, 3) and is most common in those aged 30–39 years (1). About 70% of individuals with migraine report significant impairment in daily activities (2), and approximately 20% experienced work absences due to headaches over 3 months (3).

The occurrence of migraine has been reported to be associated with various factors, including weather changes, sleep disturbances, medications, smoking, alcohol, stress, and fatigue (4). Other reported triggers include menstruation, volatile organic compounds, certain foods and beverages, and synthetic alkaloids, making the condition multifactorial (5). Among these, the most frequently reported factors are stress, menstrual cycle changes, weather changes, sleep disturbances, alcohol, and specific foods (6).

Several studies have explored the association between weather change and migraine. However, the findings remain controversial, and no clear consensus has been reached. Weather-related factors reported to be associated with migraine include atmospheric pressure, wind force, temperature fluctuations (7), average wind speed (8), humidity and warm climate conditions (9), weather changes, sunlight, cold wind, temperature increase (10), and photophobia (11). In contrast, another report concluded that the impact of weather factors on migraine and headaches is minimal or doubtful (12), while others have found that low atmospheric pressure is not a contributing factor (13, 14). As a result, there is no recognized consensus among experts, and the evidence is not considered definitive.

Populations living in regions with distinct seasonal variations are ideal for studying the relationship between seasons and migraine. Japan, characterized by pronounced seasonal change, significant temperature fluctuations, and large atmospheric pressure shifts, including typhoon effects, offers an optimal setting for such research. Despite extensive studies on weather and migraines globally, few reports have addressed the impact of weather change on migraine onset in Japan. A study in Japan investigated the relationship between migraine and atmospheric pressure using meteorological data and headache diaries from 28 patients, and the results supported an association between weather change and migraine occurrence (15). However, this study had a small sample size and was conducted in a limited region, and the impact of weather change on migraine in Japan remains unclear. A recent report using a Japanese smartphone application found that low pressure, pressure change, high humidity, and rainfall characteristic of Japan’s climate were associated with increased headache frequency (16). However, that study was limited to six regions and did not use medical record-based outcomes, such as claims data. The population included general headache sufferers, many of whom lacked a definitive diagnosis, rather than exclusively representing patients with migraine.

In this study, we aimed to investigate the impact of seasons with substantial atmospheric pressure change on the occurrence of migraine using large-scale data from a Japanese health insurance claims database matched with meteorological data.

## Materials and methods

### Study design and data source

This is a retrospective cohort study using JMDC Claims Data, which consists of health insurance claims and medical examination data from all the medical institutions in Japan (17), and national meteorological data collected by the Japan Meteorological Agency. The JMDC data enabled us to monitor all medical treatments received by the patients, even across different facilities, as long as they remained under the same health insurance. All patients in the database were younger than 75 years old and were covered by the insurance. Additionally, the Japan Weather Association offers a service called “HealthWeather®” that provides weather data such as temperature, mean atmospheric pressure, maximum and minimum atmospheric pressure, humidity, wind force, and sunlight duration. This data can be integrated with JMDC data for research.

Data from January 1, 2018, to December 31, 2019, were used. Patients were enrolled based on data from January 1, 2019, to December 31, 2019, with data from 2018 serving as the look-back period to assess eligibility.

### Study population

Patients continuously registered in the database during the study period from January 1, 2018, to December 31, 2019, with at least one diagnostic record of migraine (ICD10 code: G43) during the look-back period (January 1, 2018, to December 31, 2018) were included. Exclusion criteria included the following: (1) age under 18 years, (2) prescription records for 120 or more triptan tablets (ATC code: N02CC01, N02CC02, N02CC03, N02CC04, N02CC06) during the look-back period, (3) prescription of 120 or more ergotamine tablets during the look-back period, (4) presence of records for comorbidities associated with secondary headaches (ICD10 code; S00-S19, I60-I69, G90-G99, I11-I15, G24, and T918) during the look-back period, and (5) no prescription of triptan medication during the look-back period. Patients prescribed a total of 120 triptan tablets or more annually were excluded to rule out medication-overuse headaches. According to the Japanese headache management guidelines (18), a medication-overuse headache is defined as an “ergotamine-overuse headache” or “triptan-overuse headache” occurring on 10 or more days per month, corresponding to an annual threshold of 120 tablets.

Patients were then classified into subgroups based on the location of the healthcare facility where they were first diagnosed with migraine, corresponding to the eight regions of Hokkaido, Tohoku, Kanto, Chubu, Kinki, Chugoku, Shikoku, and Kyushu.

### Study variables

The year 2019 was divided into four seasons: winter (January–March), spring (April–June), summer (July–September), and autumn (October–December). The first day of each season was defined as the index date, and the observation period for each season extended from the index date to the last day of that season. Individuals were enrolled for each season.

Substantial atmospheric pressure change (exposure) was defined as a day when the difference between the highest and lowest atmospheric pressure in the respective region exceeded 5 hPa. We adopted this criterion based on previous research (15) and after considering several alternative definitions, as detailed in Supplementary Table S3. For each season, the number of days meeting this criterion was calculated, and the seasons with the highest and lowest atmospheric pressure change were identified.

The primary outcome of this study was the occurrence of migraine, as evaluated from the index date to the date of the first record for triptan prescription during each season’s observation period. Since this study included patients continuously registered in the database during the study period, the censor date for each season was defined as the last day of the respective season.

The following patient information was collected as covariates: sex, age, female age group, medications used for acute migraine treatment, medications used for migraine prevention, comorbidities that may trigger or exacerbate migraines, and other comorbidities. Details of the codes for medications and diseases are provided in Supplementary material. The following weather covariates were also collected: mean temperature, mean humidity, mean wind force, and sunlight duration.

### Statistical analysis

Descriptive statistics for patient characteristics are presented for each regional subgroup. Means and standard deviations (or medians and interquartile ranges) were used to describe continuous variables, while categorical variables were presented as frequencies and percentages.

For each region, the Kaplan–Meier method was used to estimate and plot the incidence-free proportion of migraine (first triptan prescription within the season), with comparison of the seasons with the highest and lowest atmospheric pressure change.

A marginal-structure Cox model was used to calculate the hazard ratio and 95% confidence intervals for the seasons with the highest atmospheric pressure change compared to the seasons with the lowest atmospheric pressure change, with time to the first triptan prescription within the season as the dependent variable. Lin and Wei’s (19) robust sandwich estimator was applied to the covariance matrix. The following covariates were included in the model: sex, age, female age group medications used for acute migraine treatment, medications used for migraine prevention, concomitant medications, comorbidities that may trigger or exacerbate migraines, and other comorbidities. Due to limited meteorological data in certain regions (data on temperature, humidity, wind speed, and sunlight duration were limited), this analysis was conducted on the entire study population without division into regional subgroups.

All analyses were conducted with SAS statistical software version 9.4 (SAS Institute Inc., Cary, NC, United States).

## Results

### Patient disposition

**Figure 1:**
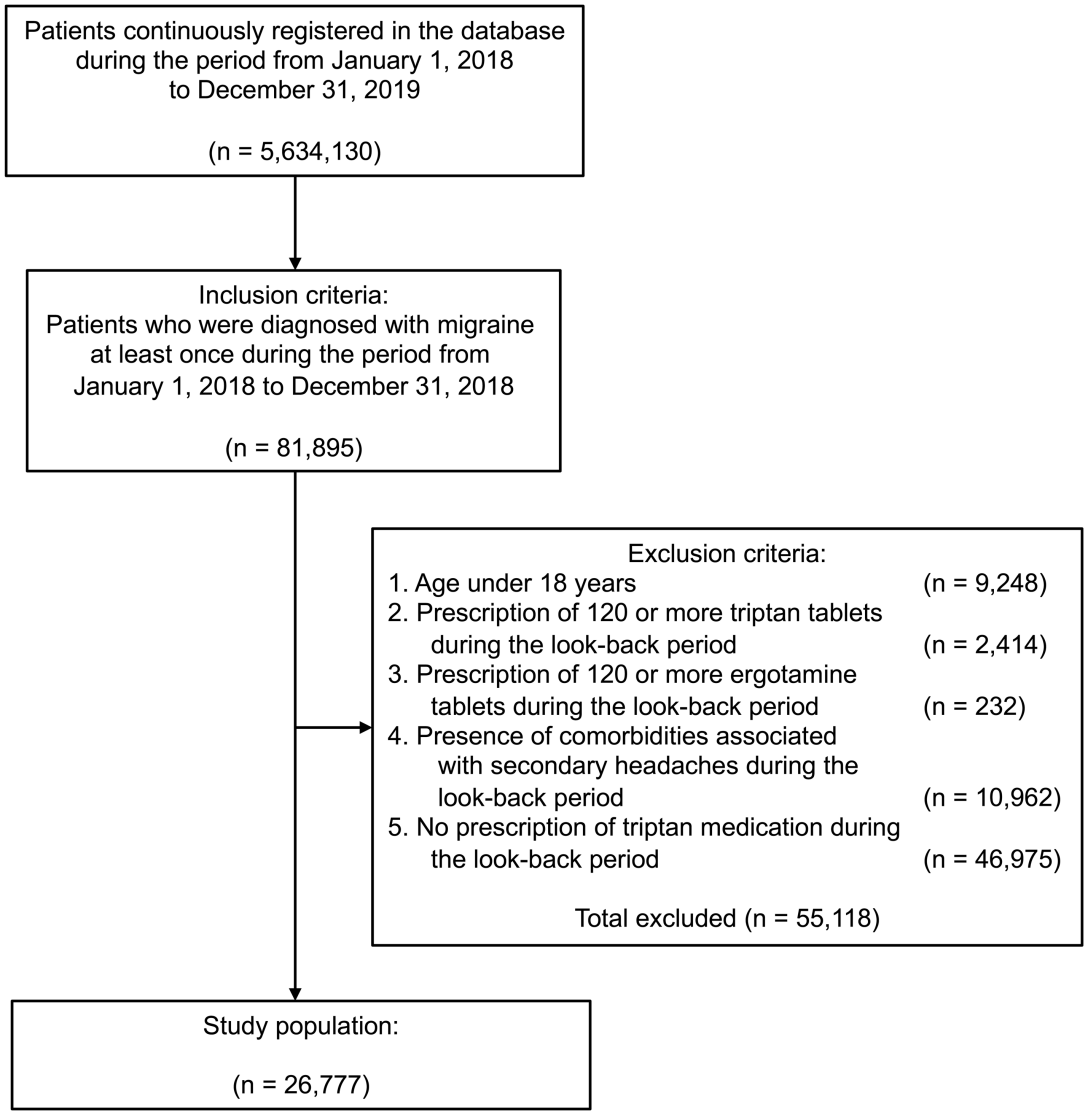
Flow diagram.

Figure 1 shows the flow diagram of the study. Of the 5,634,130 individuals enrolled in the JMDC database during the study period, 81,895 had a diagnosis record of migraine in the look-back period. After excluding patients who met the exclusion criteria, the final study population consisted of 26,777 individuals.

### Patient characteristics

Table 1 shows the patient characteristics stratified by region. The number of patients in each region was as follows: Hokkaido, 519; Tohoku, 1,495; Kanto, 10,868; Chubu, 7,235; Kinki, 3,312; Chugoku, 1,381; Shikoku, 366; and Kyushu, 1,601. Mean age ranged from 40.2 years (SD 11.5) in Hokkaido to 42.2 years (SD 11.2) in Kanto, with all regions showing a concentration of mean age in the early 40s. The percentage of females ranged from 66.9% in Chubu to 75.7% in Shikoku, with all regions showing a majority of females. The proportion of patients with comorbidities that could trigger migraines ranged from 27.1% in Kinki to 32.2% in Shikoku. Specifically, sleep disorders ranged from 13.1% in Tohoku to 17.4% in Chugoku; mood disorders from 10.2% in Tohoku to 14.4% in Kanto; anxiety disorders from 7.0% in Chubu to 10.8% in Tohoku; and dysmenorrhea from 5.8% in Kyushu to 10.4% in Hokkaido. The proportion of patients using migraine prophylactic medications ranged from 32.5% in Shikoku to 39.5% in Hokkaido. The proportion of patients using NSAIDs ranged from 22.2% in Chugoku to 28.7% in Shikoku.

**Table 1:** Patient characteristics of the study population.

Region	*N* = 26,777															
	Hokkaido		Tohoku		Kanto		Chubu		Kinki		Chugoku		Shikoku		Kyushu	
Total	519		1,495		10,868		7,235		3,312		1,381		366		1,601	
Sex, *n* (%)*																
Male	148	28.5	452	30.2	3,273	30.1	2,398	33.1	949	28.7	444	32.2	89	24.3	433	27.0
Female	371	71.5	1,043	69.8	7,595	69.9	4,837	66.9	2,363	71.3	937	67.8	277	75.7	1,168	73.0
Age, mean (SD)	40.2	11.5	40.4	11.4	42.2	11.2	41.6	11.2	42.0	11.4	41.7	11.3	40.7	11.3	41.4	11.0
Female age group, *n* (%)*																
Male or under 52 years	449	86.5	1,317	88.1	9,191	84.6	6,192	85.6	2,798	84.5	1,186	85.9	311	85.0	1,363	85.1
Female and equal or over 52 years	70	13.5	178	11.9	1,677	15.4	1,043	14.4	514	15.5	195	14.1	55	15.0	238	14.9
Comorbidities that may trigger or exacerbate migraines, *n* (%)*	153	29.5	409	27.4	3,331	30.6	1,970	27.2	899	27.1	429	31.1	118	32.2	451	28.2
Sleep disorder	74	14.3	196	13.1	1,741	16.0	989	13.7	458	13.8	240	17.4	57	15.6	221	13.8
Mood disorder	60	11.6	152	10.2	1,565	14.4	945	13.1	374	11.3	191	13.8	52	14.2	187	11.7
Anxiety disorder	49	9.4	161	10.8	1,074	9.9	508	7.0	252	7.6	111	8.0	39	10.7	148	9.2
Dysmenorrhea	54	10.4	113	7.6	707	6.5	436	6.0	217	6.6	91	6.6	24	6.6	93	5.8
Hypothyroidism	0	0.0	0	0.0	0	0.0	0	0.0	0	0.0	0	0.0	0	0.0	0	0.0
Photosensitivity	0	0.0	0	0.0	2	0.0	2	0.0	1	0.0	0	0.0	0	0.0	2	0.1
Other comorbidities, *n* (%)*	77	14.8	193	12.9	1,624	14.9	902	12.5	462	13.9	171	12.4	58	15.8	206	12.9
Primary Hypertension	56	10.8	132	8.8	1,090	10.0	688	9.5	328	9.9	123	8.9	45	12.3	165	10.3
Restless Legs Syndrome	3	0.6	1	0.1	43	0.4	43	0.6	17	0.5	9	0.7	1	0.3	6	0.4
Asthma	1	0.2	29	1.9	17	0.2	19	0.3	15	0.5	8	0.6	0	0.0	2	0.1
Epilepsy	26	5.0	40	2.7	614	5.6	193	2.7	131	4.0	40	2.9	14	3.8	45	2.8
Medication used for acute treatment of migraine, *n* (%)*	519	100.0	1,495	100.0	10,868	100.0	7,235	100.0	3,312	100.0	1,381	100.0	366	100.0	1,601	100.0
Triptan	519	100.0	1,495	100.0	10,868	100.0	7,235	100.0	3,312	100.0	1,381	100.0	366	100.0	1,601	100.0
Anxiolytics, Antipsychotics, Anesthetics, Antiemetics	181	34.9	486	32.5	3,401	31.3	2,223	30.7	1,115	33.7	540	39.1	124	33.9	544	34.0
Acetaminophen, NSAIDs	418	80.5	1,124	75.2	8,364	77.0	5,534	76.5	2,542	76.8	1,098	79.5	291	79.5	1,286	80.3
Ergotamine	4	0.8	16	1.1	112	1.0	73	1.0	28	0.8	10	0.7	2	0.5	11	0.7
Steroids	42	8.1	127	8.5	968	8.9	830	11.5	223	6.7	118	8.5	45	12.3	91	5.7
Others	59	11.4	101	6.8	919	8.5	551	7.6	278	8.4	119	8.6	21	5.7	138	8.6
Medication used for migraine prevention, *n* (%)*	205	39.5	574	38.4	4,181	38.5	2,523	34.9	1,203	36.3	538	39.0	119	32.5	582	36.4
Anti-CGRP Antibodies	0	0.0	0	0.0	0	0.0	0	0.0	0	0.0	0	0.0	0	0.0	0	0.0
Anti-CGRP Receptor Antibodies	0	0.0	0	0.0	0	0.0	0	0.0	0	0.0	0	0.0	0	0.0	0	0.0
Antiepileptic Drugs	26	5.0	123	8.2	1,239	11.4	564	7.8	259	7.8	137	9.9	30	8.2	204	12.7
Antidepressants	57	11.0	134	9.0	1,147	10.6	693	9.6	301	9.1	135	9.8	36	9.8	138	8.6
β Blockers	9	1.7	16	1.1	236	2.2	135	1.9	72	2.2	31	2.2	16	4.4	39	2.4
Calcium Channel Blockers	79	15.2	284	19.0	1,379	12.7	928	12.8	483	14.6	204	14.8	30	8.2	188	11.7
ARB/ACE Inhibitors	17	3.3	21	1.4	214	2.0	117	1.6	62	1.9	24	1.7	5	1.4	26	1.6
Other Concomitant Medications	88	17.0	154	10.3	1,551	14.3	945	13.1	422	12.7	205	14.8	36	9.8	233	14.6
NSAID	121	23.3	382	25.6	2,745	25.3	1,810	25.0	826	24.9	306	22.2	105	28.7	376	23.5

^*^Percentages were calculated using the total number of patients in each group as the denominator.

### Seasons with substantial atmospheric pressure change and migraine occurrence

**Figure 2:**
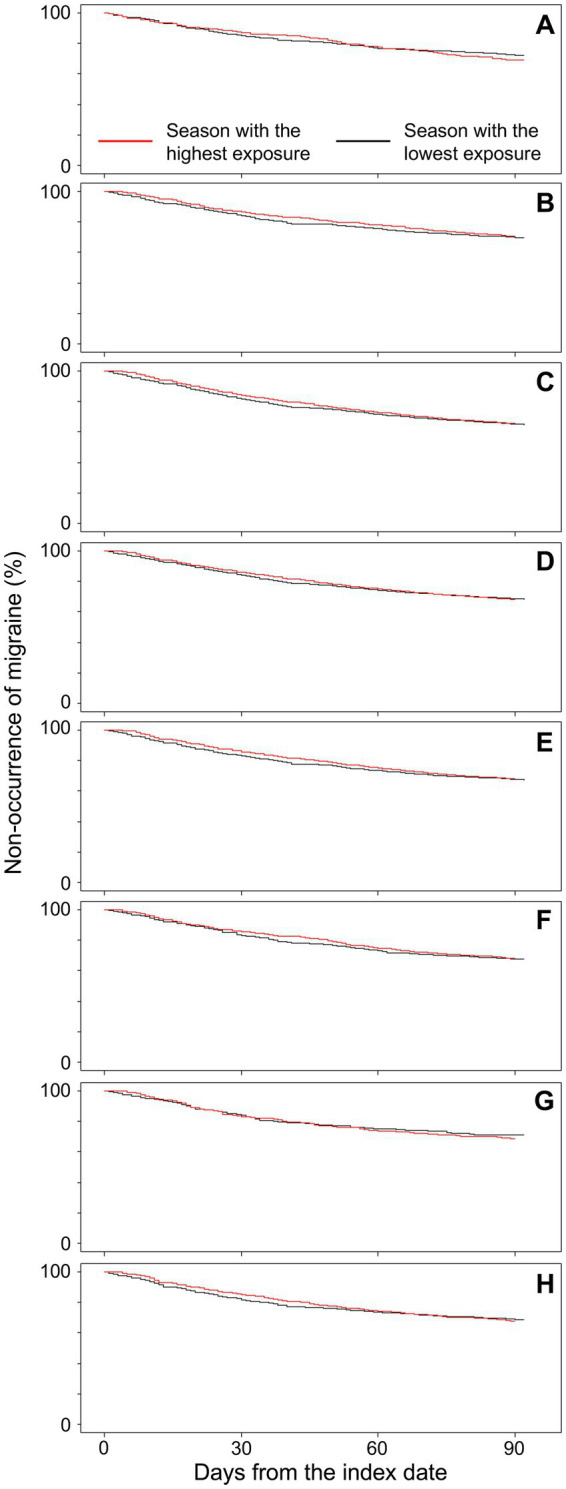
Kaplan Meier plots for the time from the index date to migraine onset (first triptan prescription within the season). **(A)** Hokkaido (*n* = 519), **(B)** Tohoku (*n* = 1,495), **(C)** Kanto (*n* = 10,868), **(D)** Chubu (*n* = 7,235), **(E)** Kinki (*n* = 3,312), **(F)** Chugoku (*n* = 1,381), **(G)** Shikoku (*n* = 366), and **(H)** Kyushu (*n* = 1,601).

Figure 2 shows Kaplan–Meier curves for the time from the index date to migraine onset (first triptan prescription within the season), stratified by the season of highest and lowest exposure in each region. In all eight regions, summer was the season with the lowest atmospheric pressure change. In Hokkaido, autumn was the season with the highest atmospheric pressure change, whereas in the other seven regions, winter showed the highest atmospheric pressure change. There were no differences in survival curves between exposure groups in any region. At 90 days, the incidence-free proportion of migraine in Hokkaido was 72.3% for the season with the lowest atmospheric pressure change and 69.4% for the season with the highest atmospheric pressure change. Similarly, the proportions were 69.8 and 70.8% in Tohoku; 65.1 and 65.5% in Kanto; 68.5 and 68.3% in Chubu; 67.6 and 68.1% in Kinki; 67.7 and 68.1% in Chugoku; 71.0 and 68.9% in Shikoku; and 68.6 and 67.7% in Kyushu.

Table 2 shows the results of Cox regression analysis for the entire study population. In the minimally adjusted model (model 1), which included sex and age as covariates, the hazard ratio for the season (exposure variable) was 0.970 (95% CI: 0.951–0.989), and no association was observed between the season and outcome (time to triptan prescription) in models that did not include adjustment for other weather-related variables. In contrast, in model 8, which included sex, age, medication used for migraine prevention, comorbidities that may trigger or exacerbate migraines, other comorbidities, and female’s age group, temperature, humidity, wind force, and sunlight hours as covariates, the hazard ratio for the highest exposure season compared to the lowest exposure season was 1.294 (95% CI: 1.007–1.663). Female sex, use of migraine prophylactic medications, and the presence of comorbidities that could trigger migraines consistently showed higher hazard ratios across all models. In model 8 (the fully adjusted model), the hazard ratios were 1.469 (95% CI: 1.402–1.538), 1.353 (95% CI: 1.300–1.407), and 1.208 (95% CI: 1.159–1.258), respectively.

**Table 2:** Adjusted hazard ratios for first triptan prescription within the season: marginal-structure Cox regression analysis.

Variable	Model 1		Model 2		Model 3		Model 4		Model 5		Model 6		Model 7		Model 8	
	HR	95%CI	HR	95%CI	HR	95%CI	HR	95%CI	HR	95%CI	HR	95%CI	HR	95%CI	HR	95%CI
Season (ref: season with the lowest atmospheric pressure changes)	0.970	0.951–0.989	0.971	0.952–0.990	0.971	0.952–0.990	0.971	0.952–0.990	1.104	0.903–1.351	1.231	0.977–1.551	1.230	0.975–1.551	1.294	1.007–1.663
Sex (ref: male)	1.405	1.345–1.467	1.402	1.343–1.464	1.392	1.332–1.454	1.469	1.403–1.539	1.470	1.403–1.539	1.468	1.402–1.538	1.468	1.402–1.538	1.469	1.402–1.538
Age	1.031	1.029–1.033	1.031	1.029–1.033	1.030	1.028–1.032	1.035	1.033–1.037	1.035	1.032–1.037	1.035	1.033–1.037	1.035	1.033–1.037	1.035	1.032–1.037
Medication used for migraine prevention (ref: None)			1.433	1.380–1.487	1.355	1.302–1.409	1.352	1.300–1.407	1.353	1.300–1.408	1.353	1.300–1.407	1.353	1.300–1.407	1.353	1.300–1.407
Comorbidities that may trigger or exacerbate migraines (ref: None)					1.210	1.162–1.261	1.208	1.159–1.258	1.208	1.159–1.258	1.208	1.159–1.258	1.208	1.159–1.258	1.208	1.159–1.258
Other comorbidities (ref: None)					1.050	0.996–1.107	1.053	0.999–1.110	1.053	0.999–1.110	1.052	0.999–1.109	1.052	0.999–1.109	1.052	0.999–1.109
Female’s age group (ref: male or under 52 years)							0.812	0.765–0.863	0.812	0.765–0.863	0.813	0.765–0.863	0.813	0.765–0.863	0.813	0.765–0.863
Mean temperature									1.007	0.996–1.018	1.010	0.999–1.021	1.010	0.999–1.021	1.011	1.000–1.023
Mean humidity											1.003	1.000–1.006	1.003	1.000–1.006	1.005	1.000–1.010
Mean wind force													0.998	0.966–1.032	1.000	0.968–1.034
Mean sunshine duration															1.021	0.994–1.050

HR, hazard ratio; CI, confidence interval.

## Discussion

This study investigated the association between seasons with substantial atmospheric pressure change and migraine occurrence using a large Japanese administrative claims database and national meteorological data. The results showed no association between seasons with substantial atmospheric pressure change and migraine onset (prescription of triptan) in any of the eight regions of Japan.

One possible explanation for the results of this study was the inability to account for localized meteorological variations at a more granular residential level. Japan’s geography, characterized by its coastal location and mountainous terrain, leads to substantial weather variation even within the same prefecture. Although this study analyzed data at the prefectural level, it lacked more granular information on local weather conditions. This limitation may have contributed to the absence of an association between seasonal change and migraine occurrence. In Cox regression analysis, no association between outcome and exposure was observed in models adjusted for some covariates. In contrast, when weather conditions were included in the fully adjusted model (model 8), the season with the highest atmospheric pressure change showed a statistically significant, although modest, higher hazard for migraine occurrence compared to the season with the lowest atmospheric pressure change. This suggests that within regions with similar weather conditions, the season may be associated with migraine occurrence.

The results of this study may also be partially explained by the influence of other confounding factors. Previous studies have indicated that healthcare-seeking behavior for migraines in Japan is relatively low (1, 2), and triptan prescriptions are typically reserved for more severe migraines (20). Consequently, migraine cases defined using claims data in this study may not have captured mild migraines. Furthermore, the regional disparity in the distribution of specialists in Japan may have influenced the findings of this study by contributing to differences in treatment practices across areas. A previous study in Japan which evaluated migraines using headache diaries supported the association between atmospheric pressure and migraine, presenting findings inconsistent with the current study (15). This also suggests that differences in migraine measurement may have resulted in the current study’s inability to capture patients with milder symptoms. Furthermore, various risk factors associated with migraine have been reported, such as stress, fatigue, menstrual cycle changes, various foods, alcohol, smoking, fasting, premenstrual periods in women, or “letdown” after stress (4, 6). More recently, atopic, psychiatric, sleep, and cardiovascular conditions have also been reported as risk factors (21), as well as hormonal imbalances, genetic and epigenetic influences (22). The current study did not fully account for these factors, which indicates that residual confounding is likely present.

This study builds on previous findings regarding the association between weather conditions and migraine. In geographic regions with marked seasonal variation, such as Japan, recognizing weather change and taking preventive actions to mitigate migraine onset is essential. However, in Japan, this association has only been reported in studies with small sample sizes. This study provides additional information by investigating the association between seasons with substantial atmospheric pressure change and migraine using a large sample size database from Japan. Consistent with previous research, factors such as female sex, the use of migraine prophylactic medications, and the presence of comorbidities that may trigger migraines were also found to be associated with increased migraine risk. However, to clarify the association, further detailed study is considered necessary.

This study has several limitations. First, data on several variables associated with migraine were not available, which suggests the potential for unmeasured confounding. Furthermore, although this study was evaluated over a single year given Japan’s distinct seasonal changes, it could not account for interannual fluctuations of pressure systems, long-term trends in extreme weather events, or delayed biological effects. Second, there is a possibility of outcome misclassification regarding migraine occurrence if triptans were prescribed as preventive medication. However, before the introduction of anti-CGRP antibodies in 2021, there were no well-established preventive treatments for migraines. Therefore, during the study period in 2019, it is likely that the majority of patients were primarily receiving acute-phase treatments. While defining outcomes by triptan prescriptions helps specifically identify migraines (23), this approach limits the sensitivity by not identifying migraine patients who use other treatments. Moreover, the high reliance on over-the-counter (OTC) medications in Japan, as highlighted in previous research (1), further reduced the sensitivity of our data. Third, it is possible that the weather of some patients’ residential areas was misclassified. In this study, regions were categorized based on the location of the medical facility where the patient was treated, which means that if a patient sought care across a prefectural or regional boundary, they might have been classified into a region different from their actual residence. However, since the number of patients who seek care across regions is likely limited, any impact on the results is expected to be minimal. Furthermore, the ecological nature of the prefectural-level meteorological data inherently has a risk of misclassification. Fourth, this study utilized a database based on corporate health insurance, which excludes the elderly and individuals enrolled in the National Health Insurance system, such as the self-employed. Therefore, there are limitations in generalizing the results to the entire population of Japan.

This study, using a large Japanese administrative claims database, did not observe an association between seasons with substantial atmospheric pressure change and migraine occurrence. Although significant results were found in multivariate analyses using national-level average meteorological data, the effect was modest, and region-level examination is considered to be necessary to assess clinical relevance. Future research should consider examining more granular residential data, beyond the prefecture level, for a more detailed analysis, and employing more advanced methodologies.

## Data Availability

The data analyzed in this study is subject to the following licenses/restrictions: the data utilized in this study were provided by JMDC Inc. under a licensing agreement. Due to these restrictions, the data are not publicly accessible. For further information regarding access to the dataset, please contact JMDC. Requests to access these datasets should be directed to JMDC Inc., https://www.jmdc.co.jp/inquiry/.
