# Supplementary material for: Association between seasons with substantial atmospheric pressure change and migraine occurrence: a retrospective cohort study using Japanese claims data and meteorological data
Source: Front Neurol. 2025 Sep 10;16:1600822. doi: 10.3389/fneur.2025.1600822 (PMC12461072; doi:10.3389/fneur.2025.1600822)
Supplement: Supplementary file 1 [file Table_1.DOCX]

# **Supplementary materials**

Table S1. WHO ATC code of concomitant medications

| Medication | WHO ATC code |
| --- | --- |
| Medications used for acute migraine treatment |  |
| Triptan | N02CC01  N02CC02  N02CC03  N02CC04  N02CC06 |
| Anxiolytics, Antipsychotics, Anesthetics, Antiemetics | A03FA01  A03FA03  N05AB04  N05AA01  N05AD08  N01AX10  N05BA01 |
| Acetaminophen, NSAIDs | N02BE01  N02BA01  M01AE01 M02AA13  M01AB05 M02AA15  M01AE02 M02AA12  M01AB01  M01AB11  M01AH01  M01AB08  M01AG01  M02AA31  M01AE  M01AC05 |
| Ergotamine | N02CA01  N02CA02  N02CA51  N02CA52  N02CA72 |
| Steroids | H02AB02  H02AB09  H02AB04 |
| Others | N02AX02  N02AJ13  N02AJ14  N02AJ15  N02AJ16  A02AA  A06AD01  A06AD02  A06AD03  A06AD04  A06AD19  A12CC |
| Medications used for migraine prevention |  |
| Anti-CGRP Antibodies | N02CD02  N02CD03 |
| Anti-CGRP Receptor Antibodies | N02CD01 |
| Antiepileptic Drugs | N03AG01  N03AX11  N03AX12  N03AX14 |
| Antidepressants | N06AA09  N06CA01  N06AA10  N06AA02  N06AA04  N06AX05  N06AX03  N06AB08  N06AB05  N05AL01  N06AX21 |
| β Blockers | C07AA05  C07AA06  C07AB02  C07AB03  C07AA12 |
| Calcium Channel Blockers | N02CX  C08DA01  C08DB01  C08CA04 |
| ARB/ACE Inhibitors | C09CA06  C09AA03  C09AA02  C09CA08 |
| Other Concomitant Medications | D00783  N02CX05  A02AA  A06AD01  A06AD02  A06AD03  A06AD04  A06AD19  A12CC  A11HA04  M03BX02  N05CH01  N05AH03 |
| NSAIDs | M02AA |

Table S2. ICD10 code of comorbidities

| Comorbidities | ICD10 code |
| --- | --- |
| Comorbidities that may trigger or exacerbate migraines |  |
| Sleep Disorder | F51  G47 |
| Mood Disorder | F30  F31  F32  F33  F34  F38  F39 |
| Anxiety Disorder | F40  F41 |
| Dysmenorrhea | N94 |
| Hypothyroidism | E39 |
| Photosensitivity | L238 |
| Other comorbidities |  |
| Primary Hypertension | I10 |
| Restless Legs Syndrome | G25 |
| Asthma | G45 |
| Epilepsy | G40 |

Table S3a. Alternative definition of the substantial atmospheric pressure change (Spring)

| Season | | Spring | | | | | | | | |
| --- | --- | --- | --- | --- | --- | --- | --- | --- | --- | --- |
| Region | | Overall | Hokkaido | Tohoku | Kanto | Chubu | Kinki | Chugoku | Shikoku | Kyushu |
| Frequency of  a day when the average atmospheric pressure in the respective region changed by more than 5 hPa from the previous day | Mean | 23.6 | 20.0 | 27.8 | 29.3 | 24.6 | 24.1 | 20.4 | 23.3 | 16.3 |
|  | SD | 5.1 | - | 1.6 | 0.8 | 3.5 | 1.6 | 1.3 | 2.2 | 5.4 |
|  | Min | 4 | 20 | 25 | 28 | 19 | 22 | 19 | 21 | 4 |
|  | Q1 | 21.0 | 20.0 | 27.0 | 29.0 | 21.0 | 23.0 | 19.0 | 21.5 | 15.5 |
|  | Median | 24.0 | 20.0 | 28.5 | 29.0 | 25.0 | 24.0 | 21.0 | 23.0 | 17.0 |
|  | Q3 | 28.0 | 20.0 | 29.0 | 30.0 | 28.0 | 25.0 | 21.0 | 25.0 | 19.5 |
|  | Max | 30 | 20 | 29 | 30 | 29 | 27 | 22 | 26 | 22 |
| Frequency of  a day when the average atmospheric pressure in the respective region changed by more than 10 hPa from the previous day | Mean | 4.4 | 7.0 | 5.7 | 6.7 | 5.0 | 4.9 | 3.8 | 4.0 | 0.8 |
|  | SD | 2.2 | - | 1.0 | 0.5 | 2.2 | 0.4 | 0.4 | 0.8 | 1.0 |
|  | Min | 0 | 7 | 4 | 6 | 3 | 4 | 3 | 3 | 0 |
|  | Q1 | 3.0 | 7.0 | 5.0 | 6.0 | 4.0 | 5.0 | 4.0 | 3.5 | 0.0 |
|  | Median | 5.0 | 7.0 | 6.0 | 7.0 | 4.0 | 5.0 | 4.0 | 4.0 | 0.0 |
|  | Q3 | 6.0 | 7.0 | 6.0 | 7.0 | 5.0 | 5.0 | 4.0 | 4.5 | 2.0 |
|  | Max | 10 | 7 | 7 | 7 | 10 | 5 | 4 | 5 | 2 |
| Frequency of  a day when the difference between the highest and lowest atmospheric pressure in the respective region exceeded 5 hPa (primary exposure definition) | Mean | 39.4 | 34.0 | 49.0 | 49.0 | 47.9 | 35.7 | 32.6 | 32.5 | 26.0 |
|  | SD | 12.6 | - | 9.2 | 4.3 | 17.9 | 3.3 | 2.6 | 2.1 | 6.2 |
|  | Min | 12 | 34 | 39 | 43 | 35 | 31 | 29 | 30 | 12 |
|  | Q1 | 32.0 | 34.0 | 41.0 | 45.0 | 37.0 | 34.0 | 32.0 | 31.0 | 24.5 |
|  | Median | 36.0 | 34.0 | 49.0 | 49.0 | 38.0 | 35.0 | 32.0 | 32.5 | 28.0 |
|  | Q3 | 45.0 | 34.0 | 58.0 | 53.0 | 50.0 | 40.0 | 34.0 | 34.0 | 30.0 |
|  | Max | 79 | 34 | 58 | 55 | 79 | 40 | 36 | 35 | 31 |
| Frequency of  a day when the difference between the highest and lowest atmospheric pressure in the respective region exceeded 10 hPa | Mean | 9.7 | 9.0 | 12.3 | 14.1 | 13.2 | 9.4 | 5.8 | 7.5 | 3.8 |
|  | SD | 4.3 | - | 0.8 | 1.2 | 3.8 | 1.3 | 0.8 | 2.5 | 1.8 |
|  | Min | 0 | 9 | 12 | 13 | 9 | 8 | 5 | 5 | 0 |
|  | Q1 | 6.0 | 9.0 | 12.0 | 13.0 | 11.0 | 8.0 | 5.0 | 6.0 | 3.0 |
|  | Median | 11.0 | 9.0 | 12.0 | 14.0 | 12.0 | 9.0 | 6.0 | 7.0 | 4.0 |
|  | Q3 | 13.0 | 9.0 | 12.0 | 15.0 | 13.0 | 11.0 | 6.0 | 9.0 | 5.0 |
|  | Max | 21 | 9 | 14 | 16 | 21 | 11 | 7 | 11 | 6 |

Table S3b. Alternative definition of the substantial atmospheric pressure change (Summer)

| Season | | Summar | | | | | | | | |
| --- | --- | --- | --- | --- | --- | --- | --- | --- | --- | --- |
| Region | | Overall | Hokkaido | Tohoku | Kanto | Chubu | Kinki | Chugoku | Shikoku | Kyushu |
| Frequency of  a day when the average atmospheric pressure in the respective region changed by more than 5 hPa from the previous day | Mean | 11.2 | 19.0 | 15.5 | 16.0 | 11.8 | 9.9 | 7.6 | 8.5 | 7.1 |
|  | SD | 4.0 | - | 1.4 | 0.8 | 2.8 | 2.5 | 0.9 | 1.3 | 2.0 |
|  | Min | 4 | 19 | 14 | 15 | 8 | 8 | 7 | 7 | 4 |
|  | Q1 | 8.0 | 19.0 | 15.0 | 15.0 | 10.0 | 8.0 | 7.0 | 7.5 | 5.5 |
|  | Median | 10.0 | 19.0 | 15.0 | 16.0 | 12.0 | 10.0 | 7.0 | 8.5 | 8.0 |
|  | Q3 | 15.0 | 19.0 | 16.0 | 17.0 | 14.0 | 10.0 | 8.0 | 9.5 | 8.0 |
|  | Max | 19 | 19 | 18 | 17 | 16 | 15 | 9 | 10 | 10 |
| Frequency of  a day when the average atmospheric pressure in the respective region changed by more than 10 hPa from the previous day | Mean | 1.3 | 3.0 | 1.0 | 1.0 | 0.6 | 0.0 | 2.4 | 1.8 | 2.4 |
|  | SD | 1.0 | - | 0.0 | 0.0 | 0.5 | 0.0 | 0.9 | 0.5 | 0.7 |
|  | Min | 0 | 3 | 1 | 1 | 0 | 0 | 1 | 1 | 1 |
|  | Q1 | 1.0 | 3.0 | 1.0 | 1.0 | 0.0 | 0.0 | 2.0 | 1.5 | 2.0 |
|  | Median | 1.0 | 3.0 | 1.0 | 1.0 | 1.0 | 0.0 | 3.0 | 2.0 | 2.5 |
|  | Q3 | 2.0 | 3.0 | 1.0 | 1.0 | 1.0 | 0.0 | 3.0 | 2.0 | 3.0 |
|  | Max | 3 | 3 | 1 | 1 | 1 | 0 | 3 | 2 | 3 |
| Frequency of  a day when the difference between the highest and lowest atmospheric pressure in the respective region exceeded 5 hPa (primary exposure definition) | Mean | 18.7 | 29.0 | 25.3 | 23.7 | 22.2 | 14.3 | 12.6 | 13.8 | 14.1 |
|  | SD | 7.4 | - | 2.4 | 2.0 | 12.0 | 1.1 | 0.5 | 1.9 | 1.6 |
|  | Min | 11 | 29 | 22 | 21 | 13 | 13 | 12 | 11 | 11 |
|  | Q1 | 14.0 | 29.0 | 23.0 | 22.0 | 16.0 | 13.0 | 12.0 | 12.5 | 13.5 |
|  | Median | 15.0 | 29.0 | 26.0 | 24.0 | 16.0 | 14.0 | 13.0 | 14.5 | 14.5 |
|  | Q3 | 23.0 | 29.0 | 27.0 | 25.0 | 21.0 | 15.0 | 13.0 | 15.0 | 15.0 |
|  | Max | 45 | 29 | 28 | 27 | 45 | 16 | 13 | 15 | 16 |
| Frequency of  a day when the difference between the highest and lowest atmospheric pressure in the respective region exceeded 10 hPa | Mean | 3.0 | 6.0 | 2.3 | 1.6 | 2.4 | 2.7 | 4.2 | 4.3 | 3.8 |
|  | SD | 1.4 | - | 1.0 | 0.5 | 1.5 | 1.0 | 0.4 | 0.5 | 1.3 |
|  | Min | 0 | 6 | 1 | 1 | 0 | 2 | 4 | 4 | 2 |
|  | Q1 | 2.0 | 6.0 | 2.0 | 1.0 | 1.0 | 2.0 | 4.0 | 4.0 | 2.5 |
|  | Median | 3.0 | 6.0 | 2.0 | 2.0 | 3.0 | 2.0 | 4.0 | 4.0 | 4.0 |
|  | Q3 | 4.0 | 6.0 | 3.0 | 2.0 | 4.0 | 4.0 | 4.0 | 4.5 | 5.0 |
|  | Max | 6 | 6 | 4 | 2 | 4 | 4 | 5 | 5 | 5 |

Table S3c. Alternative definition of the substantial atmospheric pressure change (Autumn)

| Season | | Autumn | | | | | | | | |
| --- | --- | --- | --- | --- | --- | --- | --- | --- | --- | --- |
| Region | | Overall | Hokkaido | Tohoku | Kanto | Chubu | Kinki | Chugoku | Shikoku | Kyushu |
| Frequency of  a day when the average atmospheric pressure in the respective region changed by more than 5 hPa from the previous day | Mean | 29.3 | 39.0 | 43.8 | 43.6 | 32.3 | 24.1 | 23.0 | 20.3 | 14.5 |
|  | SD | 11.2 | - | 0.8 | 1.6 | 4.1 | 0.9 | 2.9 | 1.9 | 6.0 |
|  | Min | 1 | 39 | 43 | 41 | 27 | 23 | 20 | 19 | 1 |
|  | Q1 | 20.0 | 39.0 | 43.0 | 42.0 | 29.0 | 24.0 | 21.0 | 19.0 | 13.5 |
|  | Median | 27.0 | 39.0 | 44.0 | 44.0 | 31.0 | 24.0 | 22.0 | 19.5 | 17.0 |
|  | Q3 | 42.0 | 39.0 | 44.0 | 45.0 | 36.0 | 24.0 | 25.0 | 21.5 | 17.5 |
|  | Max | 45 | 39 | 45 | 45 | 38 | 26 | 27 | 23 | 19 |
| Frequency of  a day when the average atmospheric pressure in the respective region changed by more than 10 hPa from the previous day | Mean | 7.8 | 15.0 | 14.3 | 16.6 | 9.0 | 5.0 | 3.0 | 3.3 | 0.6 |
|  | SD | 6.0 | - | 1.5 | 0.8 | 3.5 | 0.0 | 1.0 | 1.0 | 0.9 |
|  | Min | 0 | 15 | 12 | 16 | 5 | 5 | 2 | 2 | 0 |
|  | Q1 | 3.0 | 15.0 | 13.0 | 16.0 | 5.0 | 5.0 | 2.0 | 2.5 | 0.0 |
|  | Median | 5.0 | 15.0 | 15.0 | 16.0 | 10.0 | 5.0 | 3.0 | 3.5 | 0.0 |
|  | Q3 | 15.0 | 15.0 | 15.0 | 17.0 | 11.0 | 5.0 | 4.0 | 4.0 | 1.5 |
|  | Max | 18 | 15 | 16 | 18 | 14 | 5 | 4 | 4 | 2 |
| Frequency of  a day when the difference between the highest and lowest atmospheric pressure in the respective region exceeded 5 hPa (primary exposure definition) | Mean | 50.1 | 68.0 | 67.3 | 64.6 | 57.4 | 43.3 | 44.2 | 40.8 | 28.1 |
|  | SD | 15.4 | - | 2.0 | 1.7 | 12.4 | 1.5 | 3.8 | 2.6 | 10.2 |
|  | Min | 5 | 68 | 65 | 62 | 45 | 41 | 38 | 37 | 5 |
|  | Q1 | 41.0 | 68.0 | 66.0 | 63.0 | 48.0 | 42.0 | 44.0 | 39.0 | 26.0 |
|  | Median | 46.0 | 68.0 | 67.0 | 65.0 | 51.0 | 43.0 | 45.0 | 41.5 | 31.0 |
|  | Q3 | 65.0 | 68.0 | 69.0 | 66.0 | 65.0 | 45.0 | 46.0 | 42.5 | 34.0 |
|  | Max | 77 | 68 | 70 | 67 | 77 | 45 | 48 | 43 | 38 |
| Frequency of  a day when the difference between the highest and lowest atmospheric pressure in the respective region exceeded 10 hPa | Mean | 15.7 | 29.0 | 33.5 | 28.9 | 16.7 | 9.0 | 7.2 | 7.5 | 3.3 |
|  | SD | 11.3 | - | 2.7 | 2.0 | 5.7 | 0.6 | 2.8 | 1.9 | 1.6 |
|  | Min | 0 | 29 | 30 | 27 | 9 | 8 | 3 | 5 | 0 |
|  | Q1 | 7.0 | 29.0 | 31.0 | 27.0 | 11.0 | 9.0 | 6.0 | 6.0 | 3.0 |
|  | Median | 10.0 | 29.0 | 33.5 | 28.0 | 16.0 | 9.0 | 8.0 | 8.0 | 3.0 |
|  | Q3 | 28.0 | 29.0 | 36.0 | 31.0 | 22.0 | 9.0 | 9.0 | 9.0 | 4.5 |
|  | Max | 37 | 29 | 37 | 32 | 25 | 10 | 10 | 9 | 5 |

Table S3d. Alternative definition of the substantial atmospheric pressure change (Winter)

| Season | | Winter | | | | | | | | |
| --- | --- | --- | --- | --- | --- | --- | --- | --- | --- | --- |
| Region | | Overall | Hokkaido | Tohoku | Kanto | Chubu | Kinki | Chugoku | Shikoku | Kyushu |
| Frequency of  a day when the average atmospheric pressure in the respective region changed by more than 5 hPa from the previous day | Mean | 32.1 | 30.0 | 37.2 | 42.0 | 35.7 | 31.3 | 29.6 | 28.5 | 19.8 |
|  | SD | 7.5 | - | 1.8 | 1.0 | 3.1 | 1.4 | 2.5 | 2.9 | 4.4 |
|  | Min | 10 | 30 | 35 | 41 | 32 | 29 | 26 | 25 | 10 |
|  | Q1 | 28.0 | 30.0 | 35.0 | 41.0 | 34.0 | 30.0 | 28.0 | 26.5 | 19.5 |
|  | Median | 32.0 | 30.0 | 37.5 | 42.0 | 35.0 | 32.0 | 31.0 | 28.5 | 20.0 |
|  | Q3 | 38.0 | 30.0 | 39.0 | 43.0 | 38.0 | 32.0 | 31.0 | 30.5 | 22.5 |
|  | Max | 43 | 30 | 39 | 43 | 41 | 33 | 32 | 32 | 24 |
| Frequency of  a day when the average atmospheric pressure in the respective region changed by more than 10 hPa from the previous day | Mean | 6.7 | 9.0 | 7.8 | 13.1 | 7.9 | 5.6 | 4.2 | 5.0 | 2.3 |
|  | SD | 3.6 | - | 0.4 | 0.4 | 2.0 | 0.5 | 0.8 | 0.8 | 1.7 |
|  | Min | 0 | 9 | 7 | 13 | 6 | 5 | 3 | 4 | 0 |
|  | Q1 | 4.0 | 9.0 | 8.0 | 13.0 | 6.0 | 5.0 | 4.0 | 4.5 | 1.0 |
|  | Median | 6.0 | 9.0 | 8.0 | 13.0 | 8.0 | 6.0 | 4.0 | 5.0 | 2.0 |
|  | Q3 | 8.0 | 9.0 | 8.0 | 13.0 | 8.0 | 6.0 | 5.0 | 5.5 | 4.0 |
|  | Max | 14 | 9 | 8 | 14 | 12 | 6 | 5 | 6 | 4 |
| Frequency of  a day when the difference between the highest and lowest atmospheric pressure in the respective region exceeded 5 hPa (primary exposure definition) | Mean | 63.0 | 58.0 | 69.2 | 77.4 | 72.9 | 65.1 | 53.8 | 56.3 | 42.5 |
|  | SD | 13.3 | - | 6.4 | 2.0 | 8.6 | 1.8 | 6.4 | 8.2 | 6.5 |
|  | Min | 31 | 58 | 61 | 75 | 64 | 62 | 44 | 45 | 31 |
|  | Q1 | 54.0 | 58.0 | 61.0 | 76.0 | 66.0 | 64.0 | 54.0 | 50.5 | 39.5 |
|  | Median | 65.0 | 58.0 | 72.5 | 77.0 | 71.0 | 65.0 | 54.0 | 58.0 | 42.5 |
|  | Q3 | 74.0 | 58.0 | 74.0 | 79.0 | 77.0 | 67.0 | 55.0 | 62.0 | 46.0 |
|  | Max | 90 | 58 | 74 | 81 | 90 | 67 | 62 | 64 | 53 |
| Frequency of  a day when the difference between the highest and lowest atmospheric pressure in the respective region exceeded 10 hPa | Mean | 18.9 | 19.0 | 24.2 | 32.7 | 24.2 | 16.1 | 12.2 | 12.8 | 6.6 |
|  | SD | 9.5 | - | 2.6 | 1.1 | 8.9 | 0.7 | 1.5 | 2.6 | 2.3 |
|  | Min | 2 | 19 | 21 | 31 | 15 | 15 | 10 | 9 | 2 |
|  | Q1 | 12.0 | 19.0 | 22.0 | 32.0 | 18.0 | 16.0 | 12.0 | 11.0 | 5.5 |
|  | Median | 16.0 | 19.0 | 24.0 | 33.0 | 22.0 | 16.0 | 12.0 | 13.5 | 7.5 |
|  | Q3 | 26.0 | 19.0 | 26.0 | 34.0 | 28.0 | 17.0 | 13.0 | 14.5 | 8.0 |
|  | Max | 41 | 19 | 28 | 34 | 41 | 17 | 14 | 15 | 9 |
